# Quantitative trait loci analysis of fiber quality traits using a random-mated recombinant inbred population in Upland cotton (*Gossypium hirsutum* L.)

**DOI:** 10.1186/1471-2164-15-397

**Published:** 2014-05-24

**Authors:** David D Fang, Johnie N Jenkins, Dewayne D Deng, Jack C McCarty, Ping Li, Jixiang Wu

**Affiliations:** Cotton Fiber Bioscience Research Unit, USDA-ARS-SRRC, New Orleans, LA 70124 USA; Genetics & Precision Agriculture Research Unit, USDA-ARS, Mississippi State, Kragujevac, MS 39762 USA; Plant Science Department, South Dakota State University, Brookings, SD 57007 USA

**Keywords:** Cotton, Fiber quality traits, Microsatellite markers, Quantitative trait loci, Random-mating, Recombinant inbred lines

## Abstract

**Background:**

Upland cotton (*Gossypium hirsutum* L.) accounts for about 95% of world cotton production. Improving Upland cotton cultivars has been the focus of world-wide cotton breeding programs. Negative correlation between yield and fiber quality is an obstacle for cotton improvement. Random-mating provides a potential methodology to break this correlation. The suite of fiber quality traits that affect the yarn quality includes the length, strength, maturity, fineness, elongation, uniformity and color. Identification of stable fiber quantitative trait loci (QTL) in Upland cotton is essential in order to improve cotton cultivars with superior quality using marker-assisted selection (MAS) strategy.

**Results:**

Using 11 diverse Upland cotton cultivars as parents, a random-mated recombinant inbred (RI) population consisting of 550 RI lines was developed after 6 cycles of random-mating and 6 generations of self-pollination. The 550 RILs were planted in triplicates for two years in Mississippi State, MS, USA to obtain fiber quality data. After screening 15538 simple sequence repeat (SSR) markers, 2132 were polymorphic among the 11 parents. One thousand five hundred eighty-two markers covering 83% of cotton genome were used to genotype 275 RILs (Set 1). The marker-trait associations were analyzed using the software program TASSEL. At *p* < 0.01, 131 fiber QTLs and 37 QTL clusters were identified. These QTLs were responsible for the combined phenotypic variance ranging from 62.3% for short fiber content to 82.8% for elongation. The other 275 RILs (Set 2) were analyzed using a subset of 270 SSR markers, and the QTLs were confirmed. Two major QTL clusters were observed on chromosomes 7 and 16. Comparison of these 131 QTLs with the previously published QTLs indicated that 77 were identified before, and 54 appeared novel.

**Conclusions:**

The 11 parents used in this study represent a diverse genetic pool of the US cultivated cotton, and 10 of them were elite commercial cultivars. The fiber QTLs, especially QTL clusters reported herein can be readily implemented in a cotton breeding program to improve fiber quality via MAS strategy. The consensus QTL regions warrant further investigation to better understand the genetics and molecular mechanisms underlying fiber development.

**Electronic supplementary material:**

The online version of this article (doi:10.1186/1471-2164-15-397) contains supplementary material, which is available to authorized users.

## Background

Cotton is the most important natural fiber crop which supports a multi-billion dollar production and processing industry [1, 2]. The cotton genus (*Gossypium* L.) consists of about 45 diploid species belonging to eight genome groups (A-G and K) and 5 allotetraploid (AD) species [3, 4]. Within the *Gossypium* genus, two of the five tetraploid species (2*n* = 4*x* = 52; *G. barbadense* L. and *G. hirsutum* L.), along with two diploid species (2*n* = 2*x* = 26; *G. arboreum* L. and *G. herbaceum* L.), were independently domesticated for cotton fiber production in the last few thousand years in the New and Old World [5]. Of these four cultivated species, the tetraploid species *G. hirsutum*, also referred to as “Upland cotton”, accounts for about 95% of the global cotton production. Consequently, a great majority of world-wide cotton breeding programs have been focusing on improving Upland cotton.

With the increasing global demand for textile products, intense competition from synthetic fibers, and textile industry’s modernization by shifting to high-speed spinning technologies, the need for higher yielding Upland cotton cultivars with improved fiber quality has never been more critical [6]. However, yield increases are often negatively associated with fiber quality within Upland cotton [6–8]. This negative association between yield and fiber quality has hampered cotton breeding efforts for the improvement of multiple traits. It has been a cotton breeder’s high desire to effectively break this negative linkage. Random mating procedures have provided an important methodology to break undesirable associations and to form new combinations in several crop plants, including tobacco [9], sorghum [10], soybean [11], and oats [12]. Random mating has previously been shown to reduce correlations between traits in cotton as well [8, 13–15]. Random mating requires a considerable expenditure of time and energy. If one starts with a large diverse group of parental lines, it offers an opportunity to break up adverse linkage blocks and to form new recombinations, some of which should be superior.

Conventional cotton breeding programs, primarily relying on crossing adapted genotypes and selecting novel allele combinations based on phenotypes, have greatly contributed to the success of the cotton industry in the past century [16, 17]. However, the very complex quantitative inheritance of yield and fiber quality traits and the negative associations between them as described above require cotton breeders to develop more effective strategies in order to develop superior cultivars.

Marker-assisted selection (MAS) is one such strategy that has received great attention among plant and animal breeders during the past three decades. In cotton, many reports on mapping qualitative and quantitative traits have been published [2, 7, 17–23]. As for mapping quantitative trait loci (QTL) related to fiber quality, more than 1000 QTLs have been reported so far (recently reviewed by Said et al. [24]) [2, 17, 19–21, 25–32]. However, most of these QTLs were obtained based on the analysis of interspecific (mainly between *G. hirsutum* and *G. barbadense*) populations. These fiber QTLs have very limited values in Upland cotton breeding through a MAS strategy because: 1) favorable QTLs (alleles) from *G. barbadense* are often not present in *G. hirsutum*; 2) markers showing polymorphism between two species may be monomorphic within *G. hirsutum*, which in turn makes the markers not useful in Upland cotton breeding; 3) transfer of favorable traits from *G. barbadense* to *G. hirsutum* via inter-mating has been very difficult, and resulted in very limited success [27, 33]. In realization of these limitations, cotton scientists have been using *G. hirsutum* intra-specific populations to identify fiber QTLs [2, 17, 34, 35]. However, this approach faces a challenge of low intra-specific polymorphism in cotton. The average polymorphism rate between any two Upland cotton cultivars used so far in a bi-parental mapping project is about 4–8% as revealed by microsatellite markers [36–38]. Currently, there is no a high density *G. hirsutum* intraspecific genetic map that covers the entire genome.

To overcome this challenge, cotton scientists used a four-way cross population [29] or three-parent composite population [17] to map fiber and yield QTLs. The approach using populations involving more than two parents ensured greater genetic diversity and an increased polymorphism frequency in the mapping populations, and improved possibility of QTL analysis. In addition, research on association mapping agronomic trait QTLs using a group of varieties has been rising in cotton [2, 39, 40].

In this research, we first developed a recombinant inbred population of 550 lines that were derived from six cycles of random-mating beginning with half diallel crossing of 11 Upland cotton cultivars, followed by six generations of self-pollination. The 550 recombinant inbred lines (RILs) were planted in two years to obtain fiber quality measurements. Second, we screened 15538 simple sequence repeat (SSR) markers to identify polymorphic markers among the 11 parents. Third, we analyzed 275 RILs (Set 1) using 1582 well-distributed SSR markers. Fourth, we conducted association analysis between markers and fiber quality traits using the software package TASSEL [41] in order to identify fiber QTLs. Finally, for each fiber trait, we confirmed the marker-trait associations using the other 275 RILs (Set 2) by comparing the phenotypic data of the RILs that were grouped based on the genotypes of seven QTL-linked markers. The main objective of this research was to identify stable QTLs related to fiber quality traits. Since the 11 parents represent a diverse germplasm pool of the US cotton cultivars, marker-trait associations identified in this research should be very useful in Upland cotton breeding aimed at improving fiber quality.

## Results

### Genetic diversity of the 11 parents

We screened 15538 SSR markers for their polymorphism among the 11 parents (Table 1). Of these markers, 2132 (13.72%) were polymorphic. When comparing any two parents, the most different pair was between Acala Ultima and M240RNR (1231 markers, or 7.92%), and the least different pair was between FM966 and STV474 (710 markers or 4.56%). A UPGMA dendrogram tree that was constructed using 15538 SSR marker data based on the DICE coefficient [42] is presented in Additional file 1. As shown in this figure, the overall genetic diversity is very low even though these 11 parents were selected to represent the wide spectrum of diversity within the US cotton cultivars.

**Table 1:** **Eleven Upland cotton cultivars used for random-mated RI population development**

#	Cultivar	Original Developer
1	Acala Ultima	California Planting Cotton Seed Distributors (Shafter, CA)
2	Coker 315	Coker Pedigreed Seed Co. (Hartsville, SC)
3	Deltapine Acala 90	Delta and Pine Land Co. (Scott, MS)
4	Fibermax 966	Bayer Crop Science (Lubbock, TX)
5	M240RNR*	USDA-ARS (Mississippi State, MS)
6	Paymaster HS26	Paymaster Technologies, Inc. (Plainview, TX)
7	Phytogen PSC 355**	Phytogen Seeds (Indianapolis, IN)
8	Stoneville 474	Stoneville Pedigreed Seed Co. (Stoneville, MS)
9	Stoneville 825	Stoneville Pedigreed Seed Co. (Stoneville, MS)
10	Suregrow 747	Sure-Grow Co. (Centre, AL)
11	Tamcot Pyramid	Texas A&M University (College Station, TX)

*a root knot nematode resistant breeding line.

**developed by Mississippi Agriculture and Forestry Experiment Station (Mississippi State, MS) and licensed to Phytogen Seeds.

### Statistics and genome distribution of 1582 polymorphic SSR markers

Of the 2132 polymorphic markers that covered more than 80% of the tetraploid cotton genome according to the high density consensus map [43], we selected 1582 markers. These 1582 markers revealed 1585 codominant, 100 dominant and 884 monomorphic loci among the 11 parents (Table 2). A total of 4483 alleles were identified. The genomic distribution of the 1582 selected markers is shown in Table 3 and Additional file 2. These markers covered about 83% of the cotton genome if based on the high density consensus map [43]. However, if based on the *G. raimondii* genome sequence [1], the selected markers covered 740 Mbp which accounted for 93.6% coverage. Due to the unavailability of A genome reference sequence, we used the genetic map of tetraploid cotton as the main reference in this report. The coverage was uneven, ranging from 55% on chromosome (Chr.) 15 to 98% on Chr. 26. There were 23 gaps greater than 20 cM, and 19 of them occurred at the telomeric regions of 17 chromosomes. Chr.15 had the least (55%) coverage. There was a big gap of 80.47 cM on the long arm of Chr.15 where no single polymorphic marker was found. Possible reasons for this are discussed later (Discussion section). Alignment of the 2132 polymorphic markers against *G. raimondii* reference genome sequence did not identify any markers in this region either. We also screened five (SHIN-0598, CGR5835, HAU1058, HAU2550 and HAU3363) markers that were aligned to the telomeric region of *G. raimondii*’s Chr.02 (corresponding to Chr.01 or 15 of tetraploid cotton) based on the whole-genome marker map [44], however, none was polymorphic among the 11 parents.

**Table 2:** **Statistics of the 1582 polymorphic markers as revealed in 11 cotton cultivars**

Loci Type	#Loci	#Alleles
Codominant	1585	3499
Dominant	100	100
Monomorphic	884	884
Total	2569	4483

**Table 3:** **Genomic distribution of 1582 polymorphic SSR markers used for genotyping the 275 RILs of Set 1**

Chromosome	#Marker loci	Starts	Ends	Total cM covered by markers	Whole chromosome (cM)^*^	% of the chromosome	#Gaps larger than 20 cM (largest gap)
Chr.01	59	2.71	128.05	125.34	152.4	82%	1 (24.35)
Chr.02	49	4.8	121.8	117.00	133.96	87%	
Chr.03	80	23.61	156.51	132.90	159.4	83%	1 (23.61)
Chr.04	38	23.61	100.4	76.79	110.65	69%	1 (23.61)
Chr.05	125	6.89	129.16	122.27	141.4	86%	
Chr.06	50	33.7	156.19	122.49	154.6	79%	1 (33.7)
Chr.07	67	17.24	153.8	136.56	168.73	81%	
Chr.08	61	6.9	169.2	162.30	191	85%	2 (27.51)
Chr.09	75	25.6	125.6	100.00	146.06	68%	2 (25.6)
Chr.10	48	5.4	184.23	178.83	184.23	97%	1 (37.13)
Chr.11	75	13.34	203.74	190.40	228.17	83%	2 (24.43)
Chr.12	86	3.94	115.4	111.46	119.22	93%	
Chr.13	58	10.15	122.9	112.75	131.4	86%	
Chr.14	82	0	130.23	130.23	133.1	98%	
Chr.15	96	22.5	150.3	127.80	230.77	55%	2 (80.47)
Chr.16	75	17.6	119.5	101.90	139.53	73%	1 (20.03)
Chr.17	44	20.8	132.1	111.30	132.1	84%	1 (20.8)
Chr.18	50	6.9	114	107.10	121.05	88%	
Chr.19	116	24.54	185.54	161.00	189.9	85%	1 (24.54)
Chr.20	74	0	153.97	153.97	168.1	92%	1 (20.38)
Chr.21	72	25.75	167.8	142.05	173.91	82%	1 (25.75)
Chr.22	49	5.75	97.2	91.45	108.2	85%	
Chr.23	66	32.74	146.16	113.42	170.92	66%	2 (32.47)
Chr.24	56	7.35	152.81	145.46	173.84	84%	2 (21.03)
Chr.25	48	20	141.8	121.80	154.44	79%	1 (20.78)
Chr.26	75	0	148.98	148.98	152.7	98%	
unmapped	250						
Total	2024				4069.78	83%	

*Genetic distances were based on [43].

### RIL population structure assessment

**Figure 1:**
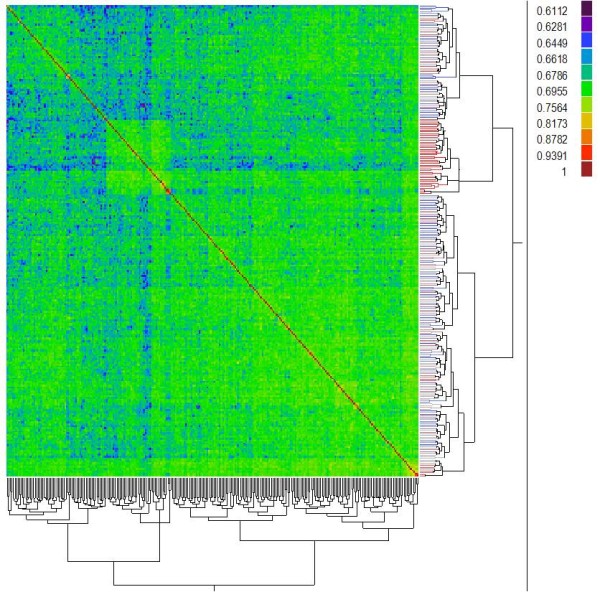
**A heat map showing the relatedness between RILs.** Relationship matrix was estimated for the relationships among the lines using marker data, which the output serves as the matrix for representing familial relatedness. The heat map displays the relationships among the 275 RILs of Set 1. The red diagonal represents perfect relationship of each line with itself, and the symmetric off-diagonal elements represent relationship measures [in this case identity by descent (IBD)] for pairs of lines. There is not an obviously block of warmer color on the diagonal which shows a cluster of closely related lines. The dendrogram (tree diagram) on the right shows the results of a cluster analysis on the IBD matrix.

The efficacy of random-mating and its effect on reducing population structure of the RIL population (Set 1) were assessed based on the 1582 polymorphic SSR markers using the software STRUCTURE 2.3 [45] and JMP Genomics 6.0 (SAS Institute, Cary, NC). These markers contained 4483 alleles (Table 2). All alleles contained in the parents were observed in the RILs. For any a given allele, its percentage within the RIL population was almost the same as that within the 11 parents (*r* = 0.99), implying thorough random-mating and uniform allele flow between generations. Furthermore, the RILs did not contain any allele that was absent in parents, indicating absence of outcrossing and successful implementation of crossing and self-pollinating. Overall, the RILs were very homozygous with average residual heterozygosity of 2.6%. Figure 1 is a heat-map generated by JMP Genomics 6.0 to show the relatedness between RILs. As seen from this figure, no two RILs appeared identical (hot color and the value as 1). Examination of the raw data indicated that RIL pairs with relatedness value higher than 0.8782 in the heat map were essentially absent. These results indicated that the random mating method employed in this study was very effective, and the population structure within the RILs essentially did not exist. Analysis of all 550 RILs using the 270 markers that were used to genotype all the 550 RILs produced the same conclusion (Additional file 3). We also assessed the RIL population structure using the software STRUCTURE 2.3, which indicated that the RIL population was thoroughly random-mated, and no obvious structure was present (Additional file 4).

### Fiber quality measurements of parents and RILs

The 11 parents and RILs were planted in triplicates in Mississippi State, MS, USA for two years (2010—2011).

The fiber properties of the RILs and their parents are shown in Table 4. Wide ranges for all traits were observed among the RILs and transgressive segregations existed. Comparisons among Pearson correlation coefficients between traits of parents, and RILs are shown in Additional file 5. The correlation coefficients were generally lower in RILs than in parents, indicating that random-mating did break linkages to a certain degree. The strong positive association between elongation (ELO) and micronaire (MIC) in parents was broken in the RILs, however, a significant positive association between bundle strength (STR) and upper-half mean fiber length (UHM) was observed in the RILs.

**Table 4:** **Fiber quality measurements of 550 RILs and the 11 parents (2010---2011 field results)**

	ELO (%)	MIC	SFC (%)	STR (g/tex)	UHM (mm)	UI (%)
RIL Mean	5.27 ± 0.15	4.60 ± 0.13	7.62 ± 0.32	29.86 ± 0.75	27.94 ± 0.51	83.48 ± 0.53
RIL Min.	3.21	3.38	6.36	24.86	24.13	80.57
RIL Max.	7.53	6.18	10.17	39.06	32.26	85.79
Parents Mean	5.62 ± 0.14	4.65 ± 0.10	7.48 ± 0.17	30.43 ± 0.46	28.19 ± 0.25	83.70 ± 0.31
Parents Min.	4.1	4.03	6.96	28.13	25.91	82.74
Parents Max.	6.96	5.11	8.03	33.23	29.72	84.66

ELO = percent elongation of fibers before breaking.

MIC = a measure of fiber fineness or maturity by an airflow instrument that measures the air permeability of a constant mass of cotton fibers compressed to a fixed volume.

SFC = short fiber content, calculated as the content (in%) of fiber shorter than 1.27 mm.

STR = force required to break a bundle of fibers one tex unit in size.

UHM = upper half mean fiber length, the average length of the longer one-half of the fibers sampled.

UI = uniformity index, calculated as the (mean length/UHM) x 100.

We analyzed the heritability of fiber traits using the 2010 and 2011 fiber measurement results of both RILs and parents. The results are shown in Table 5. The heritability (*H*^*2*^*)* of fiber traits was moderate to high ranging from 0.402 for uniformity index (UI) to 0.751 for ELO. These estimates were in agreement with those reported previously [27, 46].

**Table 5:** **Heritability of fiber traits based on the measurements of 2010 and 2011 field samples**

Fiber traits	Heritability (***H*** ^***2***^)	***p***value
ELO	0.751	<0.001
MIC	0.587	<0.001
SFC	0.438	<0.001
STR	0.628	<0.001
UHM	0.713	<0.001
UI	0.402	<0.001

### Identification of fiber QTLs

At *p* < 0.01 level, 157 marker loci were significantly associated with fiber traits, and some of them were associated with more than one trait (Table 6 and Additional file 6). When marker loci were mapped within a 5 cM interval, they were considered as a single QTL. Thus a total of 131 QTLs were identified ranging from 19 for short fiber content (SFC) to 25 for ELO (Table 6). As for chromosomal distribution, all 26 chromosomes harbored QTLs ranging from 2 (7 chromosomes) to 11 (only Chr.26). Fifty-eight QTLs were located on the A_t_ subgenome chromosomes (Chr.01-13), and 73 on the D_t_ subgenome chromosomes (Chr.14-26) (Additional file 7). A positive or negative QTL was determined based on the effects of its major allele on the trait value. If the major allele of the QTL increased a trait value, it was determined as a positive QTL. It is worth noting that a positive QTL is not necessary a favorable QTL from a breeder’s perspective for certain traits such as MIC and SFC. For each trait, there were QTLs that had positive or negative effects, and the QTL number of either type was similar. The phenotypic variance (*R*^2^ value) explained by these QTLs is listed in Table 6. The full detail is also shown in Additional file 6. Following are a brief description about these QTLs based on each trait.

**Table 6:** **Fiber QTLs identified by TASSEL and their effects**

	Total	ELO	MIC	SFC	STR	UHM	UI
#Associated marker loci	157^*^	48	43	26	26	41	38
#QTL^**^	131	25	24	19	20	20	23
Combined total QTL effects		82.8%	70.6%	62.3%	64.5%	69.4%	76.5%
#chromosomes		17	16	15	14	14	17
Positive QTL effect	No.	11	13	9	10	10	10
	*R* ^2^% mean	3.40%	2.96%	3.15%	2.44%	3.06%	3.34%
	*R* ^2^% range	1.90%--5.73%	1.44%--3.77%	1.87%--5.21%	1.60%--4.20%	1.94%--4.15%	1.82%--4.54%
	*R* ^2^% total	37.4%	38.5%	28.3%	24.4%	30.6%	33.4%
Negative QTL effect	No.	14	11	10	10	10	13
	*R* ^2^% mean	3.24%	2.92%	3.39%	4.00%	3.88%	3.32%
	*R* ^2^% range	1.86%--6.20%	1.37%--4.68%	1.15%--5.51%	2.18%--14.89%	2.44%--6.31%	1.88%--7.28%
	*R* ^2^% total	45.4%	32.1%	33.9%	40.0%	38.8%	43.2%

*some marker loci associated to more than one trait.

**marker loci mapped within 5 cM interval were considered as one QTL.

ELO: Twenty-five QTLs were identified, and located on 17 chromosomes (Additional file 7). All together, the 11 positive QTLs explained 37.4% of the total phenotypic variance with an average of 3.40% per QTL. The QTL on Chr.16 (*qELO-c16-1*, marker locus DC40054a) had the largest effect, and explained 5.73% of the phenotypic variance. The 14 negative QTLs explained a combined 45.4% of the phenotypic variance with an average of 3.24% per QTL. The QTL on Chr.05 (*qELO-c5-1*, marker locus HAU0006a) had the largest negative effect.

MIC: A total of 24 QTLs were identified, and distributed on 16 chromosomes. Chr.26 had 4 QTLs, and all were able to reduce MIC value. Thirteen QTLs had positive effects, and explained a combined 38.5% of the phenotypic variance. The QTL on Chr.20 (*qMIC-c20-2*, marker locus SHIN-0170a) had the largest effect, and could increase the MIC value by 3.77%. There were 11 QTLs that could reduce MIC value by a total of 32.1%. The QTL on Chr.18 (*qMIC-c18-1*, marker locus DPL0807a) had the largest effect, and could reduce MIC value by 4.68%.

SFC: Nineteen QTLs were identified, and located on 15 chromosomes. Nine positive QTLs explained a combined 28.3% of the phenotypic variance with an average of 3.15% per QTL. The QTL on Chr.07 (*qSFC-c7*, marker locus C2-0114a) had the largest effect, and could increase SFC value by 5.21%. A total of 33.9% of the phenotypic variance could be explained by the 10 negative QTLs. The QTL on Chr.16 (*qSFC-c16-1*, marker locus CM0066a) was responsible for 5.51% of the phenotypic variance.

STR: Twenty QTLs residing on 14 chromosomes were identified. Ten QTLs had positive effects, and explained a combined 24.4% of the phenotypic variance. The QTL on Chr.16 (*qSTR-c16*, marker locus CM0066a) had the largest effect, and was responsible for 4.10% of the phenotypic variance. Ten QTLs had negative effects, and could explain 40.0% of total phenotypic variance. One major QTL on Chr.07 (*qSTR-c7-1*, marker locus C2-0114a) had significant effect, and could decrease STR by 14.89%, the largest among all the QTLs identified.

UHM: Twenty QTLs were identified, and located on 14 chromosomes. Ten QTLs each had positive and negative effects, respectively. The largest positive effect QTL was located on Chr.18 (*qUHM-c18-1*, marker locus TMB1208b), and could increase UHM by 4.15%. The QTL on Chr.22 (*qUHM-c22-3*, marker locus HAU0086b) had the largest negative effect, and explained 6.31% of the phenotypic variance.

UI: Twenty three QTLs were found as associated with UI, and resided on 17 chromosomes. Ten QTLs could increase the UI value, and had a combined effect of 33.4% on the phenotypic variance. The QTL on Chr.16 (*qUI-c16-1*, marker locus CM0066a) had the largest effect, and was responsible for 4.54% of the phenotypic variance. Thirteen QTLs could reduce UI value by as much as 43.2% if combined. The QTL on Chr.07 (*qUI-c7-1*, marker locus C2-0114a) had the largest negative effect, and was responsible for 7.28% of the phenotypic variance.

### QTL clusters

Of the 157 marker loci associated with fiber quality traits, 37 loci were associated with more than one trait. Eighteen, thirteen, four and two were associated with 2, 3, 4 and 5 traits, respectively (Additional file 6). No single locus was associated with all 6 traits. With exception of MIC which is a complex trait (combination of fiber maturity and fineness), the effect of each QTL cluster on the traits was usually similar. In other words, a QTL cluster that was usually able to increase the values of ELO, STR, UHM or UI but reduce the SFC value is considered as a favorable QTL from a breeding point of view. An unfavorable QTL cluster usually increased SFC value but decreased the values of all other four traits. It is worth mentioning that the QTL cluster on Chr.16 (marker locus CM0066a) had the largest favorable breeding effects on these four traits (SFC, STR, UHM, UI). On the contrary, the QTL cluster on Chr.07 (marker locus C2-0114a) had the largest unfavorable breeding effects on SFC, STR, UHM and UI.

### Confirmation of fiber QTLs using the 275 RILs of Set 2

**Figure 2:**
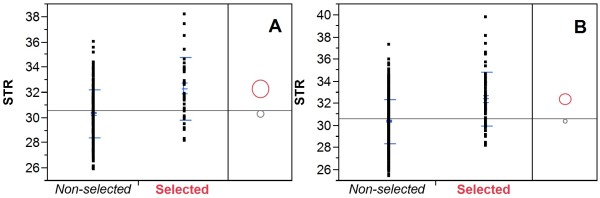
**The effect of marker selection on fiber bundle strength (STR).** Based on the genotypes of seven markers (QTLs), the 275 RILs of Set 2 **(panel A)** and all 550 RILs **(panel B)** were divided into two groups: 1) RILs containing 3 or more favorable alleles (Selected in red) and 2) the remaining RILs (Non-selected in black). The mean STR values of the selected and non-selected RILs were shown in red and black circle, respectively. The STR difference between the two groups was significant at *p* < 0.001. *Y* axis in g/tex.

One hundred seventeen SSR markers that were identified as associated with fiber traits were used to genotype the 275 RILs of Set 2. For each trait, we selected 7 markers (QTLs) to group the RILs into two groups: RILs with 3 or more favorable alleles, and the remaining RILs. We also did the same using all 550 RILs (Set 1 and Set 2). The specific markers (or QTLs associated) used for this study are listed in Additional file 6. By selecting favorable alleles based on the markers, the mean phenotypic value of the selected RILs was significantly better (higher for ELO, STR, UHM and UI, or lower for SFC and MIC) than that of non-selected RILs. Figure 2 shows the results for STR using 275 RILs of Set 2 (panel A) or 550 RILs (panel B). For the 275 RILs of Set 2, 33 RILs containing 3 or more favorable alleles at the 7 marker loci had mean fiber bundle strength of 32.32 g/tex, while 242 RILs containing fewer than three favorable alleles had mean strength of 30.34 g/tex. The difference was significant at *p* < 0.001. For the whole 550 RILs, 66 RILs met the criterion, and their mean fiber strength was 32.45 g/tex which was significant higher than the mean 30.40 g/tex of the remaining 484 RILs. Similar results were obtained for the other 5 traits (data not shown).

## Discussion

### Intraspecific genetic diversity and marker coverage

Although the 11 parental cultivars used in the present research were quite diverse in agronomic performance and breeding pedigrees, the genetic diversity among them as revealed by the 15538 SSR markers was low. These SSR markers were developed by many groups around the world. The sources of the markers were either from genomic DNA or EST sequences of many genotypes [47]. The 15538 SSR primer pairs used in screening were not pre-selected. And it is reasonable to believe that these markers cover the entire genome of tetraploid cotton as reported by Wang et al. [44]. The present research further confirms that cultivated Upland cotton has very narrow genetic diversity and close kinships possibly because of a few bottlenecks occurred during the domestication process as reported previously [3, 48–50]. In addition, there were 23 genomic regions of greater than 20 cM where no polymorphic markers were found among the 11 parents. These regions might indicate fixation (homozygosity) in the modern Upland cotton, and may contain key adaptive genes which have been fixed through breeding and selection processes. The fixed homozygous genomic blocks are a great obstacle to construct a medium to high density intraspecific *G. hirsutum* linkage map. However, it is also likely that these regions may not contain important agronomic genes or QTLs. Targeted sequencing of these regions may provide better insights about their biological functions. It is worth noting that nineteen of these 23 genomic blocks occurred at the telomeric regions of 17 chromosomes. The biological implications caused by this phenomenon remain unclear, as does their impact on practical breeding. Recently, Gore et al. [46] observed the same phenomenon when using RILs derived from a cross between TM-1 and NM24016. In our previous research to fine map the Ligon-lintless 1 (*Li*_*1*_) gene, we revealed a similar phenomenon [51], and so did Cai et al. [52]. Chr.15 had a very big gap (80.47 cM) at the end of its long arm. After examining the consensus map [43], we noticed that the region between 150 and 230 cM was almost exclusively mapped with RFLP markers. Likewise, the region between 0 and 20 cM of Chr.15 was also composed of RFLP markers in the consensus map. It is very likely that the overall genetic distance of Chr.15 may be not as long as 230 cM as reported by Blenda et al. [43]. Our marker coverage may be greater than 55%. In fact, our markers covered the range between 43,364 bp and 62,598,467 bp of the *G. raimondii* Chr.02 (corresponding to Chr.01 or Chr.15 of *G. hirsutum*) (Additional file 2), which is about 99.7% of the entire *G. raimondii* Chr.02 [1, 44]. A similar situation also exists for Chr.06, 23, 24 and 25.

### Fiber QTL numbers, chromosomal distribution and clustering

It is difficult to compare fiber QTL numbers between any two reports in the literature, because many factors affect QTL analysis results. These include experimental populations, population structure, environments, LOD threshold or significance level, and etc.. For example, Lacape et al. [19] identified 651 QTLs when using LOD 2. However, this number was drastically reduced to 167 when LOD 3.5 was used. In the present research, we used *p* < 0.01 as a threshold to declare that a marker was associated to a trait. Using such high significance threshold would without a doubt lead to some QTLs, especially those with minor effects not being identified. In fact, the combined phenotypic variance explained by the QTLs reported herein was less than 90% for all six traits (Table 6) although some of the phenotypic variance might be due to other reasons such as genotype × environment effects or epistasis. Our objective was to identify stable QTLs with moderate to major effects, and reduce the QTL number to a more manageable level from a breeder’s perspective.

The 131 fiber QTLs identified in this research were distributed on all 26 chromosomes. However, the distribution was uneven with Chr.26 harboring the greatest number of 11 QTLs. The same result was also reported by Rong et al. [21]. Chr. 26 is known to be rich in fiber genes [53], and harbors the *n*_*2*_ fiberless gene [54]. Previously, Rong et al. [21] reported that D_t_ subgenome chromosomes contained 25% more fiber QTLs than A_t_ subgenome chromosomes. A recent meta-analysis of 810 fiber QTLs [24] indicated that 23% more fiber QTLs resided on D_t_ subgenome chromosomes. Our research obtained the same conclusion showing 21% more fiber QTLs residing on the D_t_ subgenome chromosomes. Both Rong et al. [21] and Said et al. [24] reports were largely based on interspecific populations while the present study focused on an intraspecific population. Without a doubt, D_t_ subgenome chromosomes play more important roles in determining fiber quality.

**Figure 3:**
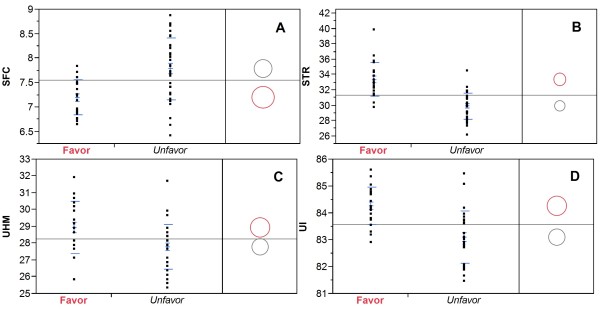
**The effect of two marker loci selection on short fiber content (SFC) (panel A), fiber bundle strength (STR) (panel B), upper half mean fiber length (UHM) (panel C) and length uniformity (UI) (panel D).** Based on the genotypes of two marker loci, i.e., C2-0114a on Chr.07 and CM0066a on Chr.16, the 275 RILs of Set 2 were divided into two groups: 1) RILs with favorable alleles at both loci (Favor in red) and 2) RILs with unfavorable alleles at both loci (Unfavor in black). The mean trait values of the two groups were shown in red and black circle, respectively. The trait value difference between the two groups was significant at *p* < 0.001. *Y* axis for SFC: %, STR: g/tex, UHM: mm, and UI: %.

QTL clustering is a common phenomenon in cotton [19, 21]. A recent meta-analysis of 1223 QTLs (fiber, yield, etc.) identified 76 QTL clusters [24]. Identification of QTL clusters will be useful in MAS since the markers delineating these regions can be used to select several traits of interests in cotton breeding. In the present research, we identified 37 QTL clusters. Two clusters, one on Chr.07 and the other on Chr.16, were particularly interesting. The QTL cluster on Chr.07 (marker locus C2-0114a) had major effects to increase SFC value but decrease the values of STR, UHM and UI. The QTL cluster on Chr.16 (marker locus CM0066a) had reverse effects. Because the directionality of the QTL effects agreed with the known phenotypic association in the parents and RIL population, it remains unclear whether this is due to the pleiotropic effects of a single QTL, or due to the co-localization or co-segregation of several fiber trait specific QTLs or genes. Chr.07 and Chr.16 are homeologous chromosomes. The chromosomal locations of these two QTLs are also comparable (58.6 cM on Chr.07 *vs* 48.3 cM on Chr.16). It is likely that they may be duplicate loci with opposite effects. Because of their large effects on multiple traits, these two QTLs serve as excellent candidates for MAS to improve fiber quality. Using our RIL population as an example, if we selected for the favorable allele of the QTLs on Chr.16 (based on the marker locus CM0066a) and against the deleterious allele of the QTLs on Chr.07 (marker C2-0114a), the four traits (SFC, STR, UHM and UI) could be simultaneously improved as compared with those selected but containing deleterious alleles for both QTLs (Figure 3). In addition, adjacent to the Chr.16 QTL cluster, there is a QTL *qELO-c16-1* (marker locus DC40054a) with a large positive effect on ELO.

### Congruence with previously reported fiber QTLs

As of today, more than 1000 fiber QTLs have been reported by many researchers. Although it is not easy to compare among the reported QTLs in different studies using different populations, it is possible to compare the QTLs identified in the present research to the prior studies using the common markers and the high density consensus genetic map. We considered two QTLs the same if the associated markers were mapped within 5 cM in the consensus map [43]. Of the 131 fiber QTLs, 77 were reported in prior researches. The detail markers and references are shown in Additional file 6. Some QTLs or clusters were reported by different research groups. Notable examples include *qUI-c3*, QTL clusters on Chr.05, Chr.07, Chr.16, and Chr.22. The fiber strength QTL, *qSTR-c24*, on Chr.24 reported by Zhang’s group [2, 25] was also identified in the present study. Most of the prior QTLs that our research agreed with were originally identified in *G. hirsutum* cultivars [2], intraspecific populations [17, 55], chromosome substitution inbred population [32], or interspecific populations but with a few generations of backcross with *G. hirsutum*[56–58]. QTLs identified in an interspecific population such as those reported by Lacape et al. or Yu et al. [7, 19, 27] agreed poorly with our QTLs. This might be due to the reasons mentioned in the Background section such as QTLs from *G. barbadense* not being present in Upland cotton. This also presents a challenge using the QTLs identified in an interspecific population in Upland cotton breeding. Fifty-four QTLs reported in this research were not found to be close to any previously-reported fiber QTLs, and could be considered as new fiber QTLs.

Said et al. [24] identified 36 fiber hotspots. We compared the fiber QTLs identified in the present research with these hotspots to see whether any of these QTLs belong to these hotspots. We used the high density consensus map and common markers as the guidance. If the QTL is within 10 cM range of the hotspot, the QTL is considered as part of the hotspot. Fourteen QTLs including 2, 7, 1, 3, and 1 for ELO, MIC, STR, UHM, and UI, respectively, likely belong to the fiber hotspots described by Said et al. [24]. The detail list of QTLs and hotspots is shown in the Additional file 6.

## Conclusions

One of the major challenges that cotton breeders have been facing when using MAS to improve quantitative traits such as fiber quality is the difficulty to transfer a QTL from other species such as *G. barbadense* into Upland cotton without compromise of other traits. While QTLs identified in an intraspecific population will have fewer obstacles to implement in breeding, the low genetic diversity within Upland cotton presents another challenge for researchers to accurately identify and map stable QTLs with moderate to high effects. In this study, we used a random-mated recombinant inbred population involving 11 diverse Upland cotton cultivars to identify fiber QTLs. This approach ensured higher polymorphism which enabled us to genotype the RIL population with 1582 polymorphic SSR markers, the most as of today. Coupled with a larger population (550 RILs, the largest single population ever reported in Upland cotton), we have identified 131 fiber QTLs and 37 QTL clusters at *p* < 0.01. Seventy-seven QTLs were mapped at the same or similar positions as previously identified QTLs in the literature, while 54 were new. By selecting favorable alleles of only 7 QTLs (markers), the mean trait value of the selected RILs could be significantly improved (higher for ELO, STR, UHM or UI, and lower for MIC or SFC). The QTL clusters on Chr.07 and Chr.16 had major effects on 4 fiber traits. Using these two loci could simultaneously improve 4 fiber traits. We are continuing to evaluate these QTLs across a panel of cotton varieties. In addition, we are testing these RILs in multiple locations to obtain yield data, and to assess the effects of random-mating on breaking the negative linkage between yield and fiber quality traits.

## Methods

### Random-mated recombinant inbred population development

Eleven Upland cotton (*G. hirsutum* L.) lines (10 cultivars and 1 breeding line) (Table 1) that represent a diverse group of non-related cotton lines from major breeding programs across the United States were chosen as parents for random mating so that the populations developed and QTLs identified should have more applicability to the entire US Cotton Belt. Pedigrees for all except M240RNR can be found in [59]. The 11 selected parents were crossed in a half diallel to produce 55 half-sib families in 2002 in Mississippi State, MS, USA. Using 55 F_1_ crosses as the founding populations, pollens from all crosses were collected, mixed, and pollinated to each founding population [60]. Random mating of the F_1_ from the half diallel was designated Cycle 0 (C_0_). Five more cycles of random-mating were made in both Mississippi State, MS, USA and the Cotton Winter Nursery in Tecoman, Mexico using the bulked pollen method [60]. These 55 families were kept separate during the random mating process. The detail of producing the random mated population was previously reported [13]. The original germplasm derived from random-mating was self-pollinated one time to increase the amount of seed for distribution and was released in 2008 under the name RMUP-C5 (Random Mated Upland Population Cycle 5) [13]. After six cycles of random mating, self-pollination was followed for six generations using single seed descent. Ten lines were randomly selected from each of these 55 founding populations and a new population including 550 RILs was created. These 550 RILs (C_5_S_6_) were used in the present research.

### Field planting and fiber quality measurement

Seeds of 550 RILs along with their 11 parents were planted as three replicates in a randomized complete block on the Plant Science Research Farm at Mississippi State, MS, in 2010--2011. Each plot was 12 m long with about 120 plants. Standard field practices were applied during the plant growing seasons. Twenty-five health-looking naturally-open bolls from the central part of a plant were hand harvested from each RIL and parent in both years. Boll samples were ginned on a 10-saw laboratory gin, and fiber samples were used for fiber property analyses. Elongation (ELO,%), micronaire (MIC), short fiber content (SFC,%), bundle strength (STR, g/tex), upper-half mean fiber length (UHM, mm), and uniformity index (UI,%) were measured by Cotton Incorporated’s fiber measurement laboratory using a High Volume Instrument (HVI, USTER Technologies Inc., Charlotte, NC). Refer to Said et al. [24] for the description of each fiber quality attribute.

### DNA extraction and SSR marker analysis

Young leaves were collected from 15 plants of each RIL, and bulked. Leaves were freeze-dried using a lyophilizer (The Virtis Company, Inc., Gardiner, NY) and crushed to powder. Total DNA was extracted from lyophilized leaf powder as previously described [22].

In order to identify polymorphic markers, we first screened 15538 SSR primer pairs using the DNAs of the 11 parents. These primer pairs were randomly selected, and their sequences were obtained from the Cotton Marker Database (http://www.cottonmarker.org). Two thousand one hundred thirty two (2132) markers were polymorphic among the 11 parents. Second, we identified the positions of these markers according to the high density consensus genetic map [43]. In addition, we used the BLASTN version 2.2.26+ algorithm to align context nucleotide sequences for 2007 of the 2132 SSR markers to the D_5_*G. raimondii* Ulbr. reference genome sequence [1] with an E-value cutoff of 1e^−20^. The clone sequences for the remainder 125 markers were not available in the public databases. Third, we selected 1582 polymorphic markers that covered as much tetraploid cotton genome as possible and were uniformly distributed across the genome. The marker selection was based on both high density genetic map [43] and sequence alignment against D_5_*G. raimondii* reference genome sequence. We also included 13 markers that were neither mapped nor had clone sequences in the public database (Additional file 2) to see whether these non-mapped markers would be as useful as mapped-markers. And fourth, we genotyped 275 RILs of Set 1 using the selected 1582 markers. These 275 RILs of Set 1 consisted of 5 randomly selected lines from each of the 55 founding populations. The other 275 RILs that were not genotyped with full 1582 markers were referring to as Set 2.

For each marker primer pair, forward primer was fluorescently-labeled at 5’ end with 6-FAM (6-carboxyfluorescein), HEX (4,7,2′,4′,5,7-hexachloro-carboxyfluorescein) or NED (7′,8’-benzo-5-fluoro 2′,4,7,-trichloro-5-carboxyfluorescein). SSR primer oligos were purchased from Sigma Genosys (Woodlands, Texas) or Life Technologies (Foster City, CA). Triplex PCR (three pairs of primers with different dyes) was performed when conducting primer screening. Hexaplex (two markers for each dye) PCR was conducted when genotyping the RILs. PCR products were separated using an ABI Genetic Analyzer 3730 xl with ROX Gene-Scan 500® as internal size standard. The PCR amplification conditions and marker data acquisition were previously described [22]. If a marker revealed two loci within the population, the duplicate marker loci were designated by adding a lower-case letter in alphabetical order after the primer name.

### Genetic diversity statistics

A pairwise matrix of genetic similarity values was calculated in NTSYSpc2.2 [61] using DICE coefficient [42]. A dendrogram tree was constructed based on Unweighted Pair Group Method with Arithmatic Mean (UPGMA) using the NTSYSpc2.2 software package.

### RIL population structure assessment

The efficacy of random-mating was estimated by evaluating the existence of potential population structure of the RILs using software packages STRUCTURE 2.3 [45] and JMP Genomics 6.0 (SAS Institute, Cary, NC). The software STRUCTURE 2.3 was implemented for a model-based clustering method for inferring population structure using 1582 polymorphic SSR marker data. Ten thousand run length was applied for the analysis to generate Q matrix, which was used for association analysis as well when using software program TASSEL [41]. When using JMP Genomics 6.0, the relatedness analysis was implemented. The relationship matrix process computes a symmetric matrix of pairwise relatedness measures for RIL entries across all SSR marker loci. Identity By Descent (IBD) was used in the study to estimate the genome-wide relatedness per JMP Genomics. The RIL population structure assessment was conducted in two ways: 1) using 275 RILs of Set 1 based on 1582 markers; 2) using 550 RILs based on 270 SSR markers.

### Phenotypic data analysis and QTL association mapping

One objective of phenotypic data analysis is to obtain precise estimate of the entry’s genetic value, where the phenotypic data were generated from the field test(s) which is confounded by environmental factors. The six fiber traits were initially screened for outliers using SAS version 9.3 software package [62] (SAS Institute, Cary, NC) by examining the Studentized deleted residuals [63] obtained from mixed linear models fitted with environment, line, and replication nested with environment as random effects. For each trait, a best linear unbiased predictor (BLUP) for each line was predicted from a mixed linear model fitted across environments with ASReml version 3.0 [64]. The statistical model is:



Where *y*_*hij*_ is an observed value, *μ* is the population mean, *E*_*n*_ is an environmental effect, *G*_*i*_ is an genotypic effect, *GE*_*hi*_ is a genotype-by-environment interaction effect, *B*_*j*(*h*)_ is a block effect, and *e*_*hij*_ is a random error. In this study, we treated all effects as random except population mean and environmental effects. This model was used to estimate variance components (including heritability) and to predict genotypic value with the following equation: . The predicted genotypic values were further used to (1) estimate the Pearson correlations among these fiber traits for parents and RIL lines, respectively, and (2) conduct association mapping and results validation as detailed in the following paragraphs. Pearson correlation analysis was conducted by SAS (SAS Institute, Cary, NC).

The association analysis for fiber QTLs was conducted using the software TASSEL 3.0 [41] by following the default protocols. We first ran a combined data analysis by a linear mixed model approach for variance component estimation and a jackknife approach for statistical test. Our results showed that GxE interaction effects were present; however, the contributions of GxE effects to these fiber traits were numerically small (ranged from 3 ~ 5%, data not shown). Thus, we used the means from the two tests for our association mapping analysis. Association analysis was first conducted using general linear model. After removing monomorphic loci and minor allele with minor allele frequency (MAF) threshold of 0.05, the Q matrix of population structure with number of estimated populations was added. Then, association analysis by using mixed linear model was implemented. In order to reduce the possibility of false QTLs, a marker locus was considered as associated with a trait only when *p* value was smaller than 0.01. A significance level was determined as following: highly significant (HS) *p* < 0.01, and extremely significant (ES) *p* < 0.002. When multiple marker loci that were mapped within a 5 cM interval were associated with a trait, they were considered as a single QTL. For the non-mapped markers, we used their physical alignments on the *G. raimondii* genome sequences to assign to a QTL. This assignment was tentative due to the difficulty to separate two homeologous chromosomes of tetraploid cotton based on only *G. raimondii* sequence. Nine non-mapped markers that were associated to a trait were not assigned to a QTL due to the uncertainty of their genetic or physical locations. In the present report, they are not included in the QTL counts, but will be included when more information becomes available. The QTL nomenclature was according to [65].

### Confirmation of fiber QTLs

Although the 275 RILs of Set 2 were not genotyped with full set of 1582 SSR markers, they were planted in the field, and phenotypic data were obtained. Based on the QTL analysis results obtained using 275 RILs of Set 1 described above, we selected a total of 117 markers to genotype the 275 RILs of Set 2 for the purpose to confirm fiber QTLs. We also genotyped the Set 2 RILs using an additional 153 SSR markers in order to assess the structure of the whole population. For each trait, we selected 7 markers (QTLs) to group the Set 2 RILs into two groups: RILs with 3 or more favorable alleles, and the remaining RILs. We also did the same using all 550 RILs.

### Availability of supporting data

The data sets supporting the results of this article are included within the article and its seven Additional files. In addition, the data about the fiber QTLs and their genomic locations reported in this article are also available at CottonGen database (http://www.cottongen.org).

## Electronic supplementary material

Additional file 1: **Genetic similarity among the 11 parents as revealed by 15538 SSR markers.** (JPEG 135 KB)

Additional file 2: **Genomic distribution of polymorphic SSR markers used for genotyping the 275 RILs of Set 1.** (XLSX 258 KB)

Additional file 3: **A heat map showing the relatedness between RILs. The heat map displays the relationships among the 550 RILs.** (DOCX 283 KB)

Additional file 4: **(A): Estimated LnP(D) over Ten repeats of STRUCTURE analysis; (B): The triangle plot of Q.** (DOCX 60 KB)

Additional file 5: **Pearson correlation coefficients among traits in random-mated RI population and parents.** (XLSX 10 KB)

Additional file 6: **Marker loci associated with fiber quality traits.** (XLSX 84 KB)

Additional file 7: **Chromosomal distributions of fiber QTLs identified by TASSEL.** (XLSX 12 KB)

## Abbreviations

Chr: Chromosome
ELO: Elongation
MAS: Marker assisted selection
MIC: Micronaire
QTL: Quantitative trait locus (loci)
RIL: Recombinant inbred lines
SFC: Short fiber content
SSR: Simple sequence repeat
STR: Fiber bundle strength
UHM: Upper-half mean fiber length
UI: Uniformity index.
