# Supplementary figures and images for: Quantitative trait loci analysis of fiber quality traits using a random-mated recombinant inbred population in Upland cotton (Gossypium hirsutum L.)
Source: BMC Genomics. 2014 May 24;15(1):397. doi: 10.1186/1471-2164-15-397 (PMC4055785; doi:10.1186/1471-2164-15-397)

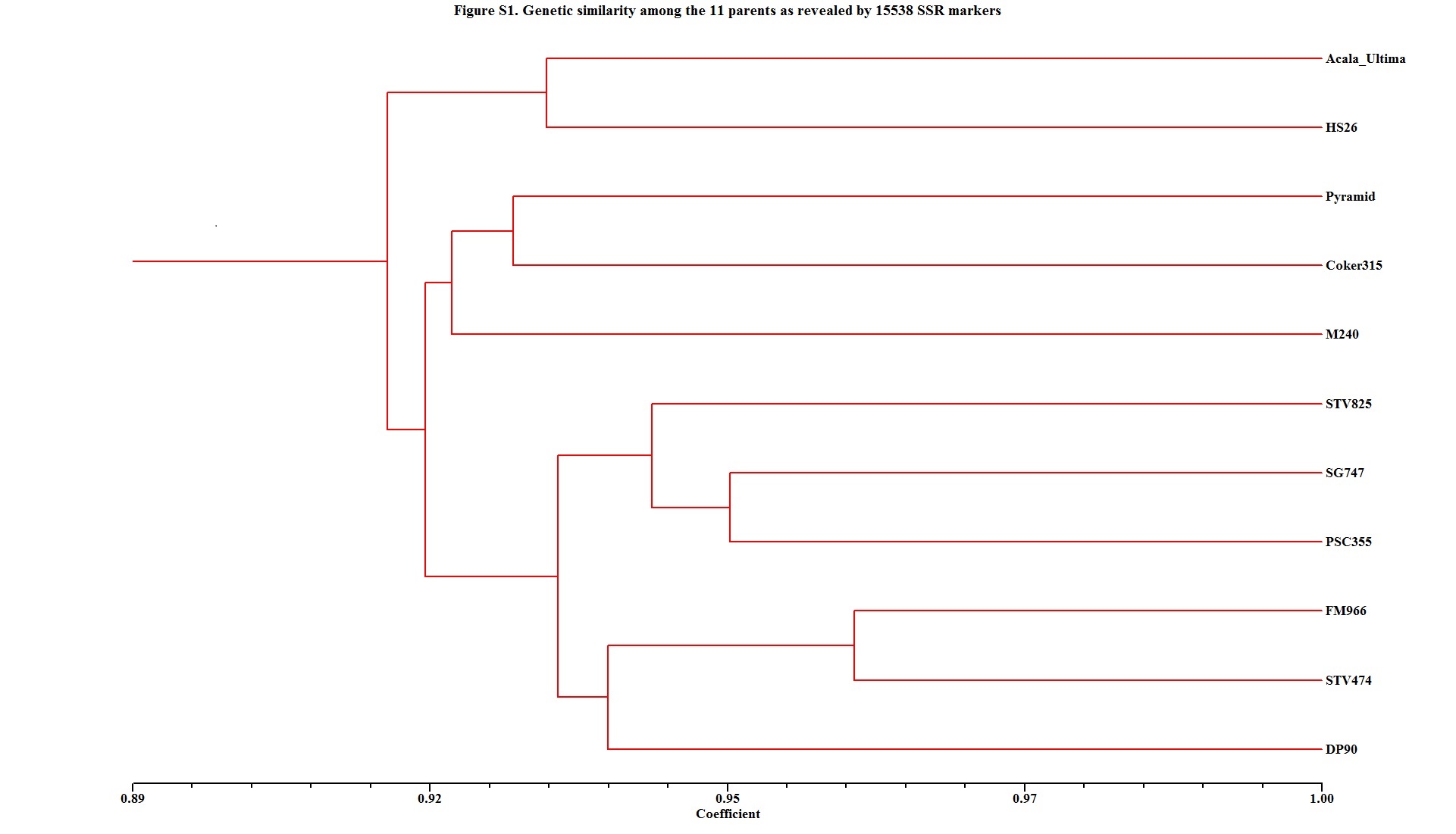

Supplement: Supplementary file 1 — Additional file 1: Genetic similarity among the 11 parents as revealed by 15538 SSR markers. (JPEG 135 KB) [file 12864_2014_6104_MOESM1_ESM.jpeg]
