# Supplementary material for: Quantitative trait loci analysis of fiber quality traits using a random-mated recombinant inbred population in Upland cotton (Gossypium hirsutum L.)
Source: BMC Genomics. 2014 May 24;15(1):397. doi: 10.1186/1471-2164-15-397 (PMC4055785; doi:10.1186/1471-2164-15-397)
Supplement: Supplementary file 3 — Additional file 3: A heat map showing the relatedness between RILs. The heat map displays the relationships among the 550 RILs. (DOCX 283 KB) [file 12864_2014_6104_MOESM3_ESM.docx]

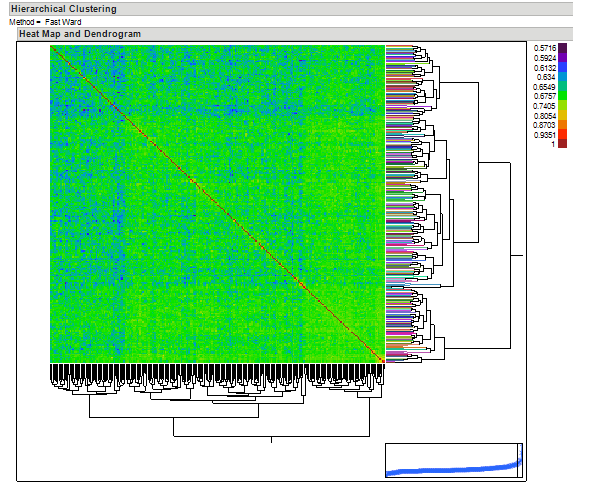


Additional file 3: A heat map showing the relatedness between RILs. Relationship matrix was estimated for the relationships among the lines using marker data, which the output serves as the matrix for representing familial relatedness. The heat map displays the relationships among the 550 RILs. The red diagonal represents perfect relationship of each line with itself, and the symmetric off-diagonal elements represent relationship measures [in this case identity by decent (IBD)] for pairs of lines. There is not an obviously block of warmer color on the diagonal which shows a cluster of closely related lines. The dendrogram (tree diagram) on the right shows the results of a cluster analysis on the IBD matrix.
