# Supplementary material for: Quantitative trait loci analysis of fiber quality traits using a random-mated recombinant inbred population in Upland cotton (Gossypium hirsutum L.)
Source: BMC Genomics. 2014 May 24;15(1):397. doi: 10.1186/1471-2164-15-397 (PMC4055785; doi:10.1186/1471-2164-15-397)
Supplement: Supplementary file 4 — Additional file 4: (A): Estimated LnP(D) over Ten repeats of STRUCTURE analysis; (B): The triangle plot of Q. (DOCX 60 KB) [file 12864_2014_6104_MOESM4_ESM.docx]

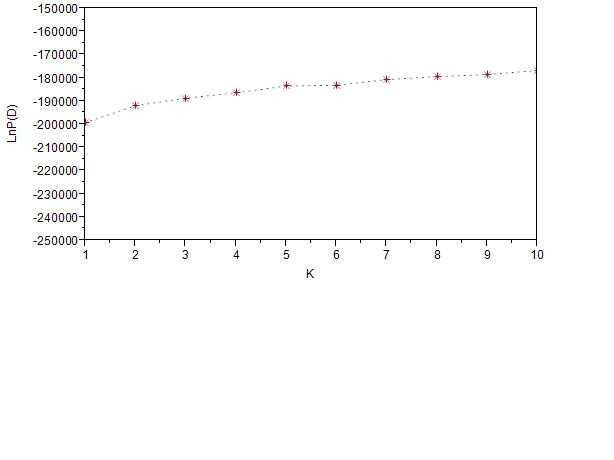


Additional file 4A. Estimated LnP(D) over Ten repeats of STRUCTURE analysis. The model-based evaluation of the population structure of the 550 Upland cotton cultivars showed that the LnP(D) value corresponding to each hypothetical k from 1 to 10 kept slightly increasing with k value and did not show any peak, which suggests the population couldn’t find a obvious structure.


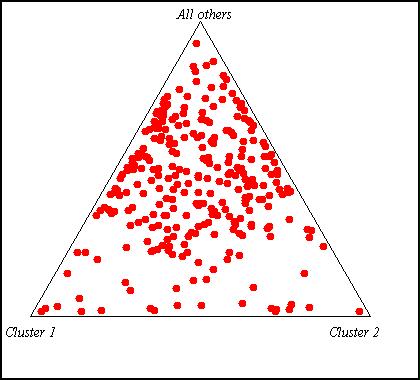


Additional file 4B: The triangle plot of Q. Each individual is represented by a colored point which is plotted onto a triangle. The colors correspond to the prior population labels. The estimated ancestry vector for an individual consists of 3 (K=3) components. For a given point, each of the three components is given by the distance to one edge of the triangle. There is not distinguished scope to separate individuals as different groups. The Run Length is 50K.
